# *Hordeum vulgare* differentiates its response to beneficial bacteria

**DOI:** 10.1186/s12870-023-04484-5

**Published:** 2023-10-04

**Authors:** Yongming Duan, Min Han, Maja Grimm, Jasper Schierstaedt, Jafargholi Imani, Massimiliano Cardinale, Marie Le Jean, Joseph Nesme, Søren J. Sørensen, Adam Schikora

**Affiliations:** 1 Julius Kühn Institute (JKI), Federal Research Centre for Cultivated Plants, Institute for Epidemiology and Pathogen Diagnostics, Messeweg 11/12, 38104 Braunschweig, Germany; 2 Leibniz Institute of Vegetable and Ornamental Crops (IGZ) - Department Plant-Microbe Systems, Theodor-Echtermeyer Weg 1, 14979 Großbeeren, Germany; 3 Institute of Environmental Biotechnology, Graz University of Technology, Petersgasse 12, Graz, 8010 Austria; 4 Institute of Phytopathology, Research Centre for BioSystems, Land Use and Nutrition, Justus Liebig University Giessen, 35392 Giessen, Germany; 5 Department of Biological and Environmental Sciences and Technologies, University of Salento, SP6 Lecce- Monteroni, Lecce, 73100 Italy; 6 Institute of Applied Microbiology, Research Centre for BioSystems, Land Use and Nutrition, Justus Liebig University Giessen, Heinrich-Buff-Ring 26-32, 35392 Giessen, Germany; 7 Laboratoire Interdisciplinaire des Environnements Continentaux (LIEC), UMR 7360 CNRS, Université de Lorraine, 8 rue du Général Delestraint, Metz, 57070 France; 8 Department of Biology, Section of Microbiology, Copenhagen University, Universitetsparken 15, Copenhagen, 2100 Denmark

**Keywords:** Induced systemic resistance, Bacterial colonization, Iron homeostasis, Seed endophytes, Barley, Rhizosphere

## Abstract

**Background:**

In nature, beneficial bacteria triggering induced systemic resistance (ISR) may protect plants from potential diseases, reducing yield losses caused by diverse pathogens. However, little is known about how the host plant initially responds to different beneficial bacteria. To reveal the impact of different bacteria on barley (*Hordeum vulgare*), bacterial colonization patterns, gene expression, and composition of seed endophytes were explored.

**Results:**

This study used the soil-borne *Ensifer meliloti*, as well as *Pantoea* sp. and *Pseudomonas* sp. isolated from barley seeds, individually. The results demonstrated that those bacteria persisted in the rhizosphere but with different colonization patterns. Although root-leaf translocation was not observed, all three bacteria induced systemic resistance (ISR) against foliar fungal pathogens. Transcriptome analysis revealed that ion- and stress-related genes were regulated in plants that first encountered bacteria. Iron homeostasis and heat stress responses were involved in the response to *E. meliloti* and *Pantoea* sp., even if the iron content was not altered. Heat shock protein-encoding genes responded to inoculation with *Pantoea* sp. and *Pseudomonas* sp. Furthermore, bacterial inoculation affected the composition of seed endophytes. Investigation of the following generation indicated that the enhanced resistance was not heritable.

**Conclusions:**

Here, using barley as a model, we highlighted different responses to three different beneficial bacteria as well as the influence of soil-borne *Ensifer meliloti* on the seed microbiome. In total, these results can help to understand the interaction between ISR-triggering bacteria and a crop plant, which is essential for the application of biological agents in sustainable agriculture.

**Supplementary Information:**

The online version contains supplementary material available at 10.1186/s12870-023-04484-5.

## Background

Microorganisms associated with plants play a key role in their fitness: some may influence the uptake of nutrients, while others may enhance resistance to biotic and abiotic challenges [1–3]. The interaction between plant defense and beneficial bacteria depends on several direct and indirect phenomena [4–6]. On the one hand, efficient colonization with beneficial bacteria of the host plant is an essential step. Beneficial bacteria can either compete with pathogens for the ecological niche (space and nutrients) or inhibit the pathogen’s growth through the secretion of antimicrobial compounds (e.g., antibiotics). On the other hand, induced systemic resistance (ISR) is a well-known, indirect phenomenon in which beneficial bacteria modulate the immunity-related gene expression of plants, thus systemically enhancing plant defenses [4, 7–11].

Pathogens and pests are the primary causes of yield losses in barley (*Hordeum vulgare*) [12], one of the main cereal crops worldwide [13]. Thus, enhanced resistance induced by beneficial plant-associated bacteria draws special attention. Several bacterial strains have been demonstrated to lessen the impact of certain barley diseases. *Pseudomonas fluorescens* MKB100 and MKB156 enhanced barley resistance against net blotch disease caused by *Pyrenophora teres* in both greenhouse and field trials [14]. Similarly, the application of *Pseudomonas chlororaphis* MA 342 reduced seed-borne diseases caused by several pathogens, including *Drechslera teres*, *Drechslera graminea*, and *Ustilago hordei* [15, 16]. *Paenibacillus polymyxa* KaI245 and *Burkholderia* sp. BE25 exhibited strong biocontrol activity against foliar diseases of barley both in vitro and in the greenhouse [17, 18]. In addition, plant systemic immunity can also be influenced by microbial secondary metabolites, such as the quorum sensing molecules *N*-acyl homoserine lactones (AHL). Resistance to both the powdery mildew-causing fungus *Blumeria graminis* f. sp. *hordei* (*Bgh*) and the leaf rust-causing fungus *Puccinia hordei* was improved in barley treated with *N*-3-oxotetradecanoyl-L-homoserine lactone (oxo-C14-HSL) or inoculated with the oxo-C14-HSL-producing bacterial strain *Ensifer meliloti* expR^+^, while this beneficial effect was absent upon inoculation with a strain impaired in AHL accumulation [19, 20]. Although beneficial bacteria confer enhanced resistance against various pathogens, the plant native response to different beneficial bacteria is still large unknown.

Understanding the gene network involved in the response to beneficial bacteria is an essential step in optimizing the effects of ISR-triggering bacteria on crop plants [21]. In *Arabidopsis thaliana*, the transcription factor MYB DOMAIN PROTEIN 72 (MYB72) plays a vital role in the early stage of ISR, induced by the root-colonizing *Pseudomonas fluorescens* WCS417r [22]. Interestingly, β-GLUCOSIDASE 42 (BGLU42), a MYB72-dependent regulator, is involved in ISR and iron-deficiency responses [23]. In addition, upon treatment with volatile organic compounds (VOCs) originating from ISR-triggering bacteria, *MYB72* and the iron uptake-related genes *FERRIC REDUCTION OXIDASE 2* (*FRO2*) and *IRON-REGULATED TRANSPORTER 1* (*IRT1*) are coregulated [24]. In Arabidopsis, IRT1 is the crucial metal transporter required for Fe uptake and proper growth; IRT1 activity influences both iron and zinc homeostasis [25]. YELLOW STRIPE LIKE 1 (YSL1) regulates the amounts of iron in seeds [26]. Several other transcription factors, such as BASIC HELIX-LOOP-HELIX TRANSCRIPTION FACTOR protein 39 (bHLH39) and BASIC HELIX-LOOP-HELIX TRANSCRIPTION FACTOR protein 47 (bHLH47), have been postulated to regulate iron homeostasis in Arabidopsis [27, 28]. In barley, genes related to the biosynthesis of flavonoids, which are associated with acquired resistance against biotic and abiotic stresses [29], were modulated by the beneficial bacterium *Acidovorax radicis* N35 [30]. Several studies indicated that the regulation of gene expression varies in response to different beneficial bacteria, hence we hypothesized that plant may differentiate its gene expression depending on the particular bacterium.

To better understand the interactions between plant hosts and beneficial bacteria, we intended to assess the impact of different plant growth-promoting bacteria (PGPB) on barley. We aimed to compare bacteria of two different origins, soil-borne and AHL-producing *E. meliloti*, with two isolates from the core seed microbiome of barley: *Pantoea* sp. and *Pseudomonas* sp. [31]. All tested bacteria enhanced plant resistance against the fungal pathogen *Bgh*, as indicated in this and in previous studies [19, 20, 31]. Reisolation experiments suggested that although all three bacteria were able to persist in the rhizosphere, they did not translocate to the phylloplane. Observation with confocal laser scanning microscopy indicated slightly different colonization patterns on roots. Very interestingly, naïve plants responded specifically to different bacterial strains. Functional analysis of the responding genes indicated that iron homeostasis, even though iron and other metal contents were not altered, was involved. Nonetheless, results gathered in this study demonstrated that plants have distinct strategies to coordinate gene regulation when they encounter different beneficial bacteria.

Recently, seed-associated microbiota have gained much attention because they may persist in successive plant generations [32, 33]. A specific core microbiome was postulated for barley seeds, present in seeds from different geographical origins, years, or genotypes [31]. Despite this, the exact composition of the barley seed microbiome seems to depend on the genotype of the parental plant [34]. Isolates of *Paenibacillus* sp., *Pantoea* sp., and *Pseudomonas* sp., obtained from the barley seed core microbiome [31], were able to colonize young barley roots, promote plant growth, and induce plant resistance against the fungal pathogen *Bgh*. Here, we wondered whether the application of beneficial bacteria would affect the composition of seed endophytes, thereby benefiting the next generation. Root inoculation with soil-borne *E. meliloti* seemed to have an impact on the composition of seed endophytes, especially in the case of *Enterobacter* and *Pantoea.* Importantly, the resistance induced by bacterial inoculation is not echoed in the next generation. In total, our study revealed several characteristics of the impact that beneficial bacteria have on barley. These factors may help to utilize ISR to reduce disease development in the field.

## Results

### Beneficial bacteria of different origins enhanced barley resistance to fungal pathogen

**Fig. 1:**
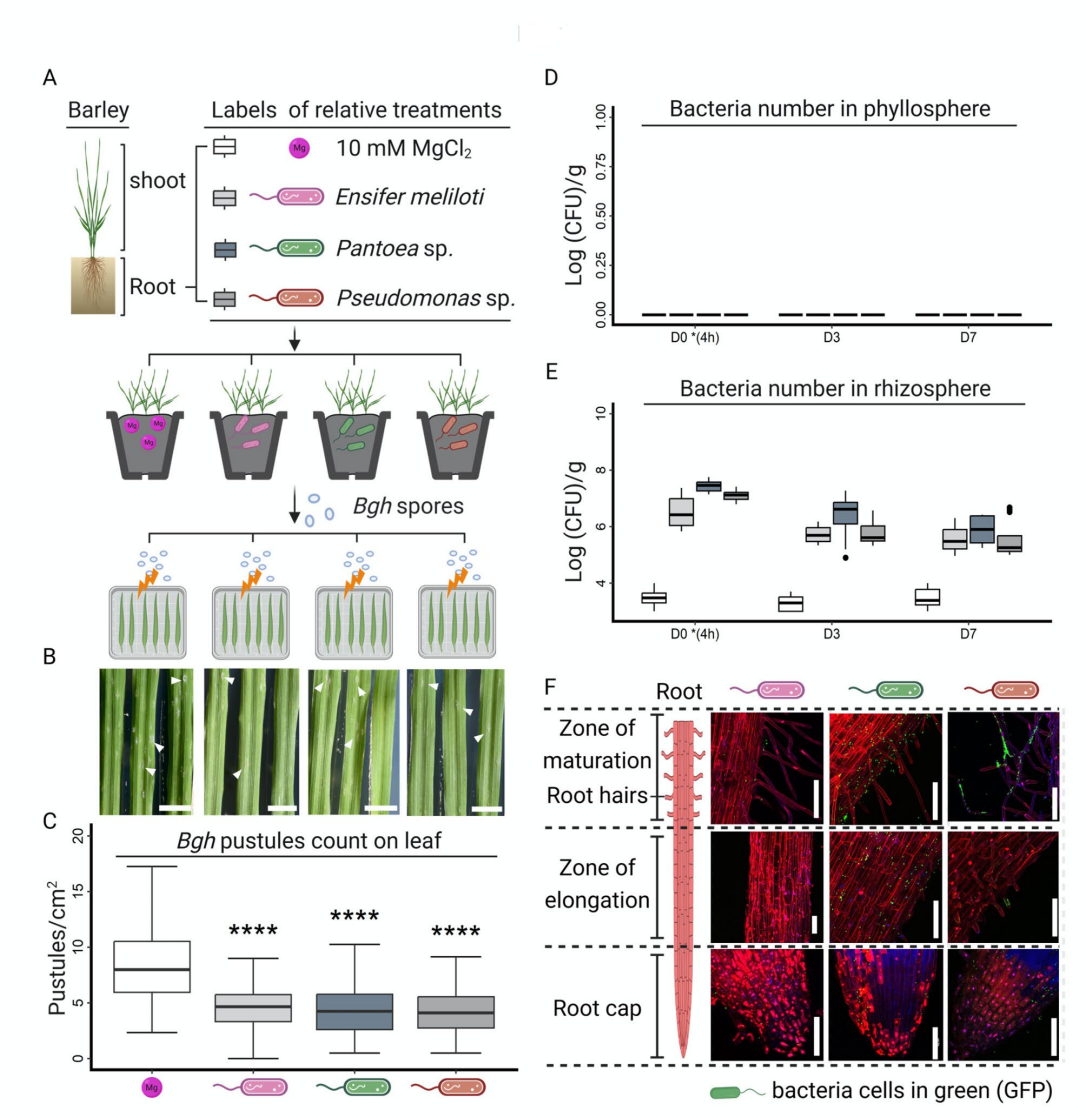
Bacteria with various root colonization patterns increased resistance against fungal pathogens. The barley rhizosphere was inoculated three times with either *E. meliloti*, *Pantoea* sp., *Pseudomonas* sp., or 10 mM MgCl_2_ as a control **(A)**. Plant resistance was assessed by counting fungal pustules in a detached leaf assay **(B)**. Arrows indicate fungal pustules on the surface of representative leaves, five days after challenge with spores of *Blumeria graminis* f.sp. *hordei* (*Bgh*), bars indicate 1 cm (**A, B**, and **C**). Statistical analysis was performed with Student’s *t*-test individually between the control and bacterial treatments, **** indicates *p* < 0.0001, *n* ≥ 130. Spontaneous rifampicin-resistant mutants of three bacteria were used to drench barley roots with the bacterial suspension. The bacteria in the shoot **(D)** and root **(E)** were enumerated at day zero (4 h after inoculation) and three and seven after drenching. The bacterial number was normalized to root or shoot weight. Each bacterial treatment contained a minimum of four biological replicates. Bacteria harboring the GFP pSM1890 plasmid were inoculated into hydroponic barley roots for three days. The images **(F)** show colonization patterns of beneficial bacteria on different root areas, including the root cap, zone of elongation, and zone of maturation, as well as root hairs. PGPB cells are shown in green (GFP), the cell walls of roots are indicated in red (PI), and the cell nuclei of roots are indicated in magenta (DAPI and PI). Bars indicate 100 μm

Three plant growth-promoting bacteria (PGPB) of different origins: the soil-borne *Ensifer meliloti* (*Sinorhizobium meliloti*) strain Rm2011, which was chosen because of its ability to produce a long-chain *N*-acyl-homoserine lactone: oxo-C14-HSL [35], and representative isolates from barley seeds *Pantoea* sp. (strain P_s_AC_ 13b) and *Pseudomonas* sp. (strain P_s_CA_4b) [31], were tested for their impact on the resistance of the barley (*Hordeum vulgare*) cultivar Golden Promise towards *Blumeria graminis* f. sp. *hordei* (*Bgh*). To this end, bacteria were cultured and drenched into greenhouse-grown barley roots three times (Fig. 1A) as described previously [19, 31, 36]. Three days after the last inoculation, plants were challenged with the fungal spores (Fig. 1A). Plant resistance was assessed by counting fungal pustules five days after the challenge in a detached leaf approach (Fig. 1A, and 1B). Compared to 10 mM MgCl_2_ inoculated plants with leaves developing approximately eight pustules/cm^2^, inoculation with *E. meliloti*, *Pantoea* sp., and *Pseudomonas* sp. significantly reduced the number of pustules to approximately five pustules/cm^2^ on leaves (Fig. 1C). The results indicated that the selected bacteria enhanced barley resistance against *Bgh*. This phenomenon was also observed in other studies [19, 31, 36].

### Bacteria were not detectable in leaves, while they persisted stably on roots with different colonization patterns

Enhanced resistance against foliar fungal pathogens is a systemic phenomenon occurring mainly on leaves, while beneficial bacteria are applied to roots. In the next step, we wondered whether the beneficial bacteria could translocate to the upper parts of plants, such as leaves (phylloplane), directly competing with fungal pathogens. To assess this, we used spontaneous rifampicin-resistant mutants. Greenhouse-grown barley plants were root-drenched with bacterial suspensions. The presence of bacterial cells was assessed in the rhizosphere and in leaves at day zero (4 h after drenching), as well as three and seven days after bacterial inoculation (Fig. 1D, and 1E). Bacterial numbers in the rhizosphere slowly decreased and reached approximately 10^6^ CFU/g root seven days after inoculation (Fig. 1E). Meanwhile, the bacteria were not detectable in leaf samples (Fig. 1D). These results demonstrated that bacteria do not translocate from the root area (rhizosphere) to the phyllosphere, even if present in the rhizosphere. Notably, a small amount (approximately 10^3^ CFU/g root) of native bacteria with rifampicin resistance was observed in the solvent (10 mM MgCl_2_) control (Fig. 1E). Even though the native rifampicin-resistant bacteria probably did not affect the CFU counts in bacterial treatments, diverse native bacteria may influence the colonization patterns of beneficial bacteria on roots.

To verify the colonization patterns, *E. meliloti*, *Pantoea* sp., and *Pseudomonas* sp. were marked with Green Fluorescent Protein (GFP) using the pSM1890 plasmid. Barley plants were grown in a sterile hydroponic system for two weeks, and then GFP-marked bacterial strains were inoculated into the root media. This setting was chosen to avoid the influence of native bacteria on the tested bacterial strains. The colonization patterns were assessed after three days. The images revealed that different areas of the root surface (rhizoplane), including the root cap, zone of elongation, and zone of maturation as well as root hairs, were colonized at different levels by the three beneficial bacteria (Fig. 1F). Cells of *E. meliloti* were found at the root cap, in the elongation zone, and in the maturation zone of the root. Many cells of *Pantoea* sp. were found on the root cap, and the bacteria colonized the elongation zone and the maturation zone in a rather homogeneous pattern. Cells of *Pseudomonas* sp. were also observed at the root cap; however, unlike *E. meliloti* or *Pantoea* sp., *Pseudomonas* sp. began to form biofilm-like structures on the root hairs in the maturation zone. These images demonstrated that the three bacteria have distinct preferences when colonizing barley roots.

### Stress- and ion-related genes responded to the different beneficial bacteria

**Fig. 2:**
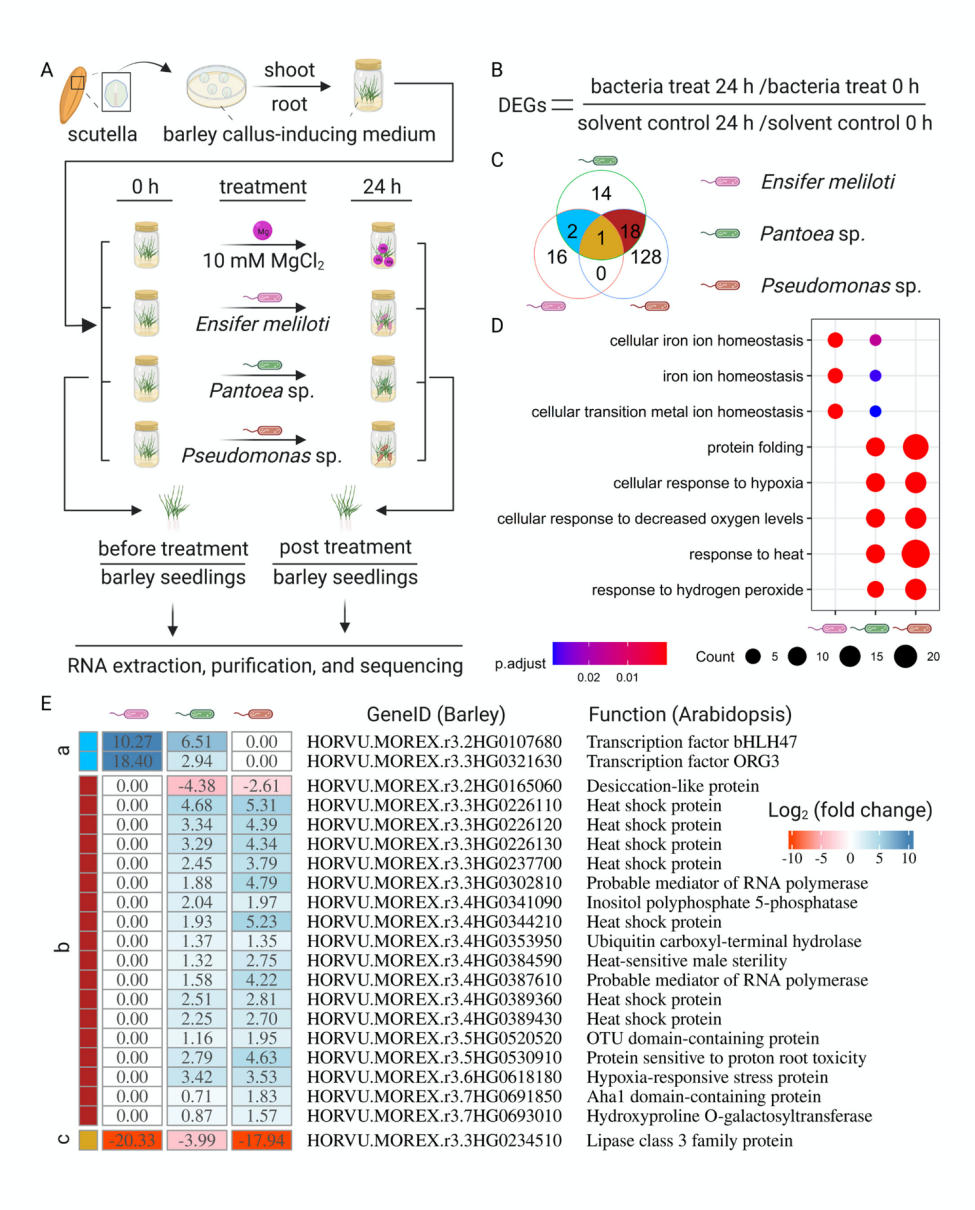
Stress- and ion homeostasis-related genes responded specifically to different bacteria. Endophyte-free barley seedlings were obtained using a callus-inducing method based on scutella from seeds **(A)**. These plants were inoculated individually with *E. meliloti*, *Pantoea* sp., *Pseudomonas* sp., or 10 mM MgCl_2_ as a control **(A)**. Whole RNA extraction, purification, and sequencing were performed using whole barley seedlings collected before and 24 h post treatment in three biologically independent replicates **(A)**. Differentially expressed genes (DEGs) were identified in the comparisons between the bacterial treatments and control at the thresholds (adjusted *p* < 0.05 and the fold change of gene expression > 1.5) **(B**, and **C)**. To further reveal the respective function, the barley gene ID was converted to the gene ID of Arabidopsis, according to the protein sequence. The converted gene ID was used to perform an enriched GO terms **(D)** analysis. The overlap of DEGs **(C)** was used to isolate commonly regulated genes, a indicates 2 genes, b indicates 18 genes, and c indicates one gene. The estimated function of commonly regulated genes in barley was predicted on the related description in Arabidopsis **(E)**

The above results indicated that the enhanced resistance of plants conferred by the tested bacteria represents induced systemic resistance (ISR), while the tested strains have different colonization patterns. We wondered therefore how the plant would respond to the different beneficial bacteria. To fully decode the first response of a naïve plant to beneficial bacteria, we performed transcriptome analysis using sterile endophyte-free barley plants available for this study. These plants were regenerated based on the callus-inducing method [37, 38], of which the initial point is the scutella of barley seeds (Fig. 2A). In addition, these plants tested negative for bacterial 16S gene amplicons prior to the experiments.

Total RNA was extracted from whole hydroponically grown sterile barley seedlings before (0 h) and 24 h after inoculation with the three tested bacteria, using 10 mM MgCl_2_ as a solvent control. Considering that variable gene expression backgrounds may exist among different plant seedlings, gene expression was compared first between samples 0 and 24 h after bacterial inoculation. Thereafter, a second comparison was performed between the bacterial treatments and the control. Differentially expressed genes (DEGs) were identified using the adjusted *p* < 0.05 and the fold change > 1.5 thresholds (Fig. 2B).

In total, 19, 35, and 147 DEGs were identified in *E. meliloti, Pantoea* sp., and *Pseudomonas* sp. inoculated plants, respectively (Fig. 2C; Supplementary Data Set S1). Of those genes, 16 genes specifically responded to the presence of *E. meliloti*, 14 genes specifically responded to *Pantoea* sp., and 128 genes were specific to inoculation with *Pseudomonas* sp. To further reveal the related functions, the barley gene ID was converted into *Arabidopsis thaliana* gene ID, according to their protein sequence. The converted gene ID was used to analyze the enrichment of GO terms. The top three GO terms enriched in response to *E. meliloti* were related to ion or inorganic ion homeostasis and cellular iron ion homeostasis (Fig. 2D). Those GO terms were also enriched in response to *Pantoea* sp., whereas the GO terms shared by inoculation with *Pantoea* sp. or *Pseudomonas* sp. were related to protein folding, cellular response to hypoxia and decreased oxygen levels, and response to heat and hydrogen peroxide (Fig. 2D).

To further investigate the function of genes commonly regulated by bacterial presence, the function of DEGs responding to different bacteria was deduced on the basis of related Arabidopsis gene ID (Fig. 2E). The two genes responding to *E. meliloti* and *Pantoea* sp. inoculation were orthologs to the genes of *bHLH39/ORG3* and *bHLH47/PYE*, which regulate the iron deficiency response in Arabidopsis [28, 39]. Furthermore, the relative gene expression level in *E. meliloti-*inoculated plants was much higher than that in *Pantoea* sp. inoculated barley. Many of the genes that responded to *Pantoea* sp. and *Pseudomonas* sp. were orthologs to genes encoding heat shock proteins, which may be involved in multiple stress responses in Arabidopsis [40]. Meanwhile, the relative gene expression level in *Pseudomonas* sp. inoculated plants is generally higher than that of *Pantoea* sp. inoculated barley. Only one gene (*HORVU.MOREX.r3.3HG0234510*) responded to the presence of all three bacteria, and it is an orthologue to a lipase class family protein. These results demonstrated that plants respond to different bacteria with a specific gene expression pattern.

### Genes related to iron homeostasis were specifically triggered by bacteria

**Fig. 3:**
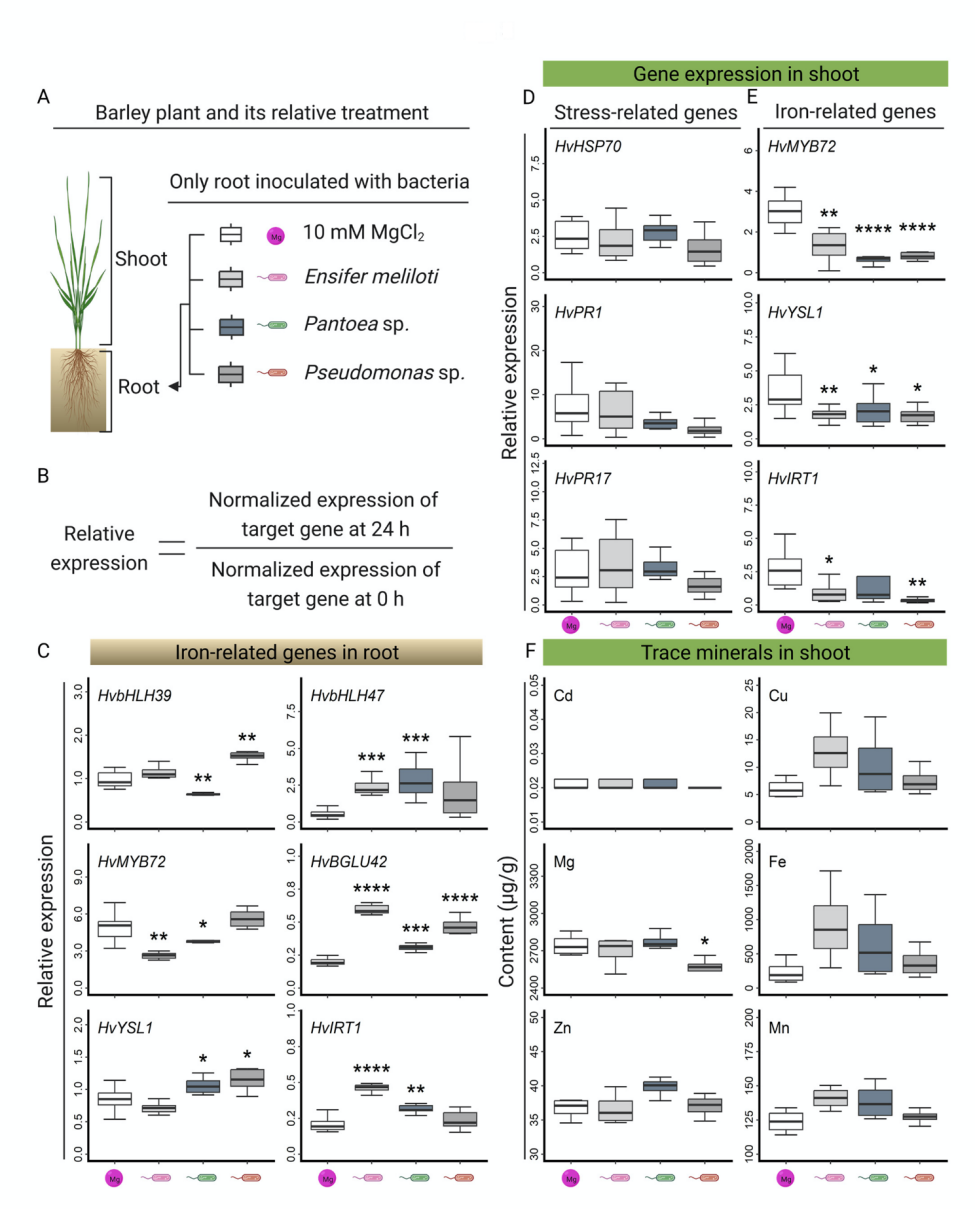
Iron homeostasis-related genes specifically respond to bacteria. Hydroponically grown barley plants were inoculated with bacterial suspension or with 10 mM MgCl_2_ as a control **(A)**. Roots and shoots were collected before and 24 h after inoculation. Orthologues of genes related to iron homeostasis in Arabidopsis were selected as candidates for quantitative RT-PCR. The expression of *HvUBQ60* was used to normalize the expression levels. Presented are the relative expressions compared to the expression level before the bacterial inoculation (**B**, **C**, **D**, and **E**). Differences between the control and bacterial inoculation were assessed with Student’s *t-*test, * indicates adjusted *p* < 0.05, ** adjusted *p* < 0.01, *** adjusted *p* < 0.001, and **** adjusted *p* < 0.0001, respectively, *n* = 4. Trace minerals, including cadmium (Cd), copper (Cu), magnesium (Mg), iron (Fe), zinc (Zn), and manganese (Mn), were determined in the shoots of hydroponically grown barley plants three days after bacterial inoculation **(F)**. Differences between the control and bacterial inoculation were assessed with Student’s *t-*test, * indicates adjusted *p* < 0.05, *n* = 4

In Arabidopsis, the expression of several genes involved in iron homeostasis, such as *IRT1*, *YSL1*, *BGLU42* and the transcription factors *MYB72*, *bHLH39*, and *bHLH47* [27, 28], was also regulated in response to beneficial bacteria. Transcriptome analysis in barley revealed that genes related to iron homeostasis specifically responded to the presence of *E. meliloti* and *Pantoea* sp. (Fig. 2D and E). To confirm our results in an independent approach, we verified the expression of the chosen genes with quantitative RT-PCR. Sterile hydroponically grown barley plants were root inoculated with bacterial suspensions or with 10 mM MgCl_2_ (Fig. 3A). Roots and shoots were collected separately before (0 h) and 24 h post treatments. Similar to the RNA-sequencing approach in this study, the relative expression of target genes was calculated by comparing the normalized expression of the target gene between 0 and 24 h to avoid the potential influences of different gene expression backgrounds in seedlings (Fig. 3B).

The candidate genes of barley were selected based on their potential relation to iron homeostasis. Protein sequences that were similar to the protein sequences encoded by iron homeostasis-related genes (*bHLH39, bHLH47, BGLU42, MYB72, YSL1*, and *IRT1* in Arabidopsis) were chosen as barley orthologs (Supplementary Data Set S1). Previous studies revealed that the genes *BGLU42* and *MYB72* function as essential nodes to coordinate the bacteria-induced ISR and iron homeostasis in the root system [23, 24]. Furthermore, stress-related genes, including *Heat Shock Protein 70* (*HvHsp70*) and defense-related genes (*HvPR1* and *HvPR17*), were also selected to determine their gene expression in the shoot.

Compared to the control, *HvbHLH39*, an orthologue of bHLH transcription factor 39, was specifically triggered in roots by inoculation with *Pseudomonas* sp. and inhibited by inoculation with *Pantoea* sp., while *HvbHLH47* significantly responded to *E. meliloti* and *Pantoea* sp. (Fig. 3C). Similar phenomena were observed in the case of orthologous genes encoding iron transporters. *HvYSL1* specifically responded to the presence of *Pantoea* sp. and *Pseudomonas* sp., while *HvIRT1* significantly responded to *E. meliloti* and *Pantoea* sp. (Fig. 3C). Interestingly, the expression of *HvBGLU42*, encoding a β-Glucosidase, was significantly stimulated by all three tested bacteria (Fig. 3C), whereas the expression of the *MYB72* ortholog gene *HvMYB72* did not respond to *Pseudomonas* sp. nor was it regulated after inoculation with *E. meliloti* and *Pantoea* sp. (Fig. 3C). Unlike the upregulated gene expression in the root, the stress-related genes, including *HvHSP70*, *HvPR1*, and *HvPR17*, in the shoot were not regulated by bacterial treatments (Fig. 3D). Surprisingly, iron-related genes were downregulated or not regulated in the shoot by bacterial treatments (Fig. 3E). Taken together, these results indicated that in barley, iron homeostasis-related genes are specifically regulated upon inoculation with beneficial bacteria.

The contrasts in the expression of iron-related genes between roots and shoots drove us to explore whether the metal content would change upon inoculation. To answer this question, roots of two-week-old hydroponically grown sterile barley plants were drenched with a bacterial suspension. Considering that ISR is a systemic phenomenon, only shoot samples were harvested 72 h after bacterial inoculation, the time point when plants were challenged with *Bgh*, and 10 mM MgCl_2_ was used as a control. The contents of six metals, including iron (Fe), cadmium (Cd), copper (Cu), magnesium (Mg), manganese (Mn), and zinc (Zn), were analyzed. We observed no differences in the concentration of the tested metals (Fig. 3F), except for Mg. The magnesium content was significantly lower in plants inoculated with *Pseudomonas* sp. The content of iron in the shoot was mildly enriched by root inoculation with *E. meliloti* and *Pantoea* sp. but not *Pseudomonas* sp. Similar slightly changed contents of Cu and Mn also fit the trend (Fig. 3F).

### Root drenching with beneficial bacteria affected the composition of seed endophytic microbiota

**Fig. 4:**
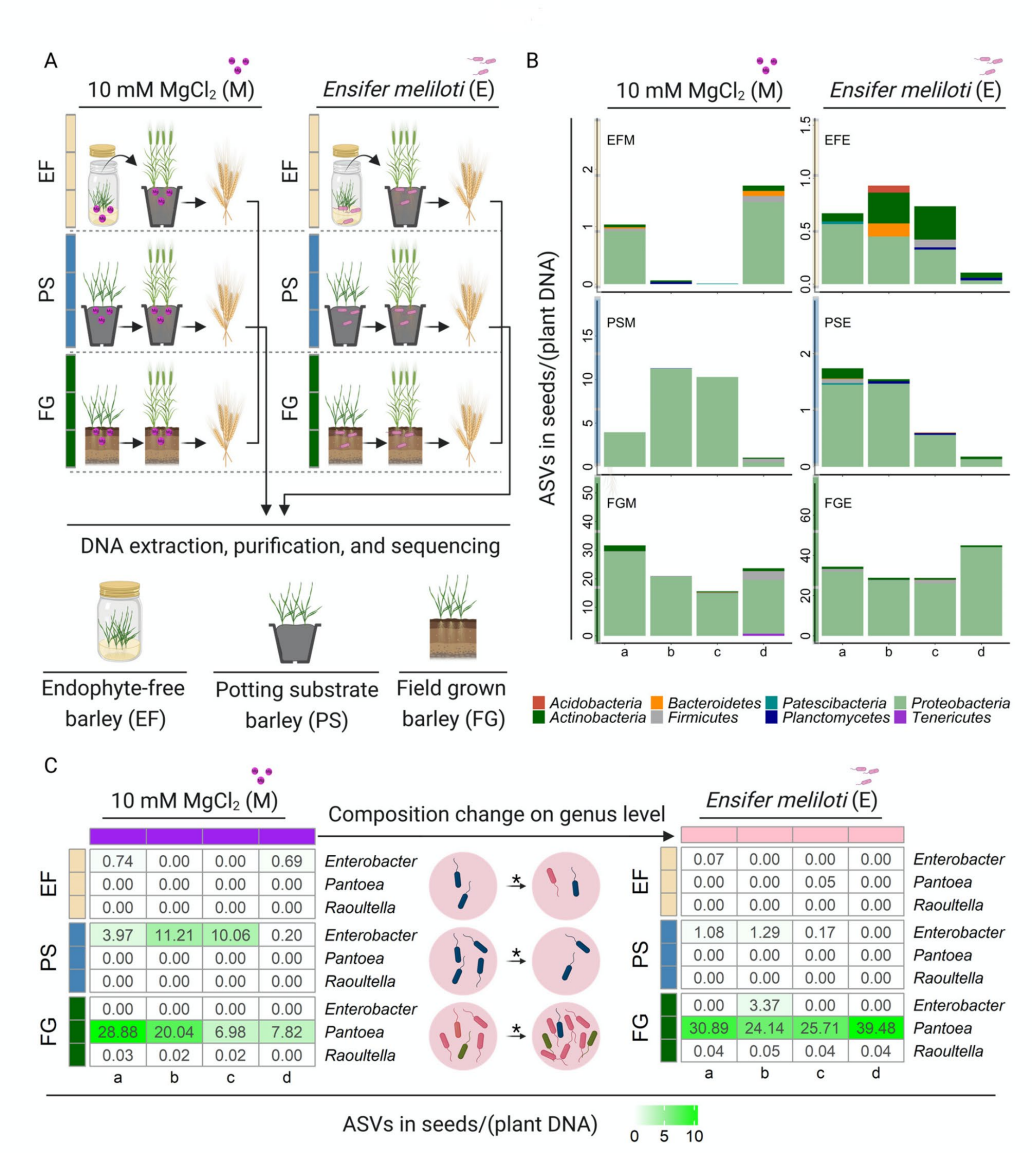
Composition of seed endophytes influenced by bacterial treatment under different growth conditions. Barley plants were grown in a greenhouse or field until seed harvest **(A)**. Seeds were collected from endophyte-free (EF), potting substrate (PS), and field-grown (FG) plants inoculated with *E. meliloti* (EFE, PSE, FGE). The sterile, in vitro-grown, endophyte-free barley plants were inoculated with bacteria three days before being transferred into potting substrate and allowed to grow until harvest in the greenhouse. Ten millimolar MgCl_2_ was used as a control (samples EFM, PSM, and FGM). Samples were harvested in quadruplicate (as indicated by a-d). The amplicon sequence variant (ASV) counts were normalized to the amount of plant DNA. The composition of the prokaryotic community is shown at the phylum level, and *Proteobacteria* was the predominant phylum in the seed endophytes **(B)**. The composition of the prokaryotic community is also shown at the genus level, and the genera of which ASV abundance changed after inoculation are listed **(C)**. Specific genera such as *Enterobacter* showed deceased abundance comparing the control to bacteria treatments if seeds were collected from endophyte-free barley and potting substrate-grown barley. However, the abundance of *Pantoea* and *Raoultella* increased when comparing the control to bacterial treatments if seeds were collected from field-grown barley. Statistical analysis was performed with Student’s *t*-test, * indicates *p* < 0.05

Seed endophytes play an important role in plant fitness, especially during the development of seedlings, which motivated us to explore the question of whether inoculation with particular bacterial strains would influence the composition of seed microbiota. To answer this question, we assessed the composition of endophytic microbiota in seeds produced by inoculated plants. However, considering German legal restrictions, such as Biostoffverordnung, while using *Pseudomonas* sp. and *Pantoea* sp., the following assay was performed only with *E. meliloti*, a model strain used in various previous studies [19, 36, 41]. Seeds were harvested from endophyte-free (EF) barley plants and potting substrate (PS) barley plants, both of which were grown under greenhouse conditions, as well as from field-grown plants (FG) (Fig. 4A). In all growing conditions, plants were inoculated with *E*. *meliloti* (EFE, PSE, and FGE) as an exemplary bacterium known to enhance barley resistance towards *Bgh*. MgCl_2_ (10 mM) was used as a solvent control (EFM, PSM, and FGM). Sequencing of the 16 S rRNA gene fragment was used to assess the microbial community (Fig. 4B). *Proteobacteria* was the predominant group in the seed endophytic microbiota under greenhouse conditions (Fig. 4B). Even though the number of amplicon sequence variants (ASVs) was very small, especially in the case of seeds originating from EF and PS plants (approximately one ASV per plant DNA), some patterns could be observed. Specific genera, such as *Enterobacter*, seem affected by bacterial treatment in seeds of greenhouse-grown barley (EF and PS), displaying a smaller number of ASVs or even zero count in seeds originating from *E. meliloti*-inoculated plants (EFE and PSE) (Fig. 4C). Seeds from field-grown barley plants were used to verify our findings. Similarly, *Proteobacteria* were the prevalent endophytic bacteria in seeds from field-grown barley (Fig. 4B). The number of ASVs related to *Pantoea* and *Raoultella* increased in seeds originating from field-grown plants after bacterial treatment with *E. meliloti* (FGE) (Fig. 4C). It must be noted that the usual methods such as principal component analysis to analyze such data were not suitable here due to the extremely small number of ASVs. These results demonstrated that bacterial inoculation influences the composition of seed endophytic microbiota.

### Induced systemic resistance was not echoed in the following generation

To explore the potential effects of the bacterial inoculants on the next generation, parental plants grown in a greenhouse were inoculated with *E. meliloti*, *Pantoea* sp., and *Pseudomonas* sp. as described above and grown until harvest under greenhouse conditions. To assess whether induced resistance could be passed to the next generation, harvested seeds were germinated, and the subsequent plants were tested for their resistance towards *Bgh*, as previously described (Fig. 1). We observed no differences between plants originating from control seeds and plants grown from seeds of which parental plants were inoculated with either of the tested bacteria (Supplementary Fig. S1 and S2). The enhanced resistance of parental plants seems lost in the following generation. These findings indicated that under the tested conditions, the beneficial effects, such as increased resistance, might be too subtle to influence plants in future generation(s).

## Discussion

From an agronomical point of view, induced resistance is of high importance since it is an effective strategy to cope with plant diseases [4, 5]. Our study focused on the bacterial colonization pattern, plant gene expression and modification of seed microbiota after inoculation with different ISR-triggering bacteria in barley.

To effectively elicit ISR, beneficial bacteria should maintain a minimal concentration at approximately 10^5^ CFU per gram root for several days [4, 9, 42]. *Pantoea* sp. and *Pseudomonas* sp. originating from barley seeds, colonized roots of young barley plants [31]. In our study, the crop plant barley was in contact with approximately 10^6^ bacterial CFU/g root, which persisted stably throughout the experiments (Fig. 1E). Interestingly, the three beneficial bacteria indeed have different colonization preferences (Fig. 1F). *Pseudomonas* sp. attached to root hairs, forming biofilm-like structures, whereas *Pantoea* sp. preferred to attach to the root surface of the mature zone. Unlike those two colonization patterns, *E. meliloti* showed a rather unspecific colonization pattern. Similarly, such spatially distinct colonization patterns were observed along developing roots for two *Populus* isolates, *Pantoea* sp. YR343 and *Variovorax* sp. CF313 [43]. The initial colonization and its pattern may also influence later interactions within the microbial community at the host-symbiont interface [44, 45].

Some beneficial bacteria, such as *Bacillus thuringiensis*, display the ability to enter root tissues and migrate through the xylem to distal plant parts, especially leaves [46]. Bacteria tested in this study were not detectable in the phyllosphere (Fig. 1D), suggesting that the root-to-leaf translocation phenomenon is not a general feature of bacteria beneficial for plants. Meanwhile, the enhanced resistance conferred by three bacteria was a systemic phenomenon since the infection with the fungal pathogen (*Bgh*) occurs predominantly on leaf and occasionally on stem surfaces. This fact rules out the possibility of direct trophic competition between a beneficial bacterium and the fungal pathogen. Furthermore, the *E. meliloti* strain used in this study produces *N*-3-oxotetradecanoyl-L-homoserine lactone (oxo-C14-HSL) as its quorum sensing molecule [35]. Both oxo-C14-HSL-producing bacteria and pure *N*-acyl homoserine lactone (AHL) molecules enhanced barley resistance against diverse pathogens. Interestingly, this AHL-priming response is dependent on the cultivar [19]. In specific cultivars such as Golden Promise used in this study, plant resistance could be stimulated by AHL molecules, while in others, such as Gaulois, plant resistance could not be stimulated. The mechanisms of perception and response to AHL molecules are not yet clear in barley. In Arabidopsis, jasmonic acid, salicylic acid and auxin play an important role in the plant response to AHL molecules [47–50]. Recently, our group postulated that AHL-Priming Protein 1 (ALI1) is essential for the AHL-priming response in Arabidopsis [51]. Similar to *E. meliloti*, *Pantoea* sp. belongs to AHL-producing bacteria (Supplementary Fig. S3) [52, 53]. The presence of AHL molecules may therefore be one possible explanation for why barley responded to *Pantoea* sp. and *E. meliloti* in a rather similar manner. In vitro and *in planta*, *Pantoea agglomerans* ZJU23 secretes the antifungal compound herbiclin A, which inhibits pathogen growth directly by binding and disrupting membrane lipid rafts [54]. In Arabidopsis, volatile organic compounds produced by *Pseudomonas fluorescens* WCS417 can induce the expression of systemic immunity-related genes [24]. Antifungal or volatile organic compounds may also be secreted by the bacteria used in this study [55, 56], which offers another possible explanation for the beneficial impact. Interestingly, even though the original sources of the beneficial bacteria are different (soil-borne *E. meliloti* or seed endophytes *Pantoea* sp. and *Pseudomonas* sp. ), the level of triggered ISR was similar (Fig. 1C). Thus, an investigation on the common and divergent characteristics of bacteria triggering ISR would be helpful in the quest for new approaches in agriculture, as well as in our understanding of plant-bacteria interactions.

Like all other organisms, crop plants need to perceive and distinguish between beneficial and pathogenic microorganisms. Transcriptome analysis of endophyte-free barley encountering the tested bacteria for the first time revealed differences in gene expression. Compared to the control, *E. meliloti*, *Pantoea* sp., and *Pseudomonas* sp. triggered changes in the expression of different genes, which indicates that barley can respond differently to distinct bacteria. Heat shock-related genes mainly responded to *Pantoea* sp. and *Pseudomonas* sp. Although the heat shock protein-encoding gene *HvHSP70* was not regulated by inoculation with bacteria in this study (Fig. 3D), the extensive overlap between the heat and biotic/abiotic stress response pathways indicates that heat shock-related proteins might be involved in plant immunity [40], whereas iron homeostasis-related genes responded to inoculation with *E. meliloti* and *Pantoea* sp.

Recent studies in dicotyledon plants revealed an overlap between the plant immune response and iron deficiency response [7, 57–60]. To the best of our knowledge, this phenomenon has not been described until now in monocotyledons, such as barley. Our study suggests that genes related to iron homeostasis respond to beneficial bacteria (Figs. 2 and 3). We observed an enrichment of iron homeostasis-related GO terms, as well as the enhanced expression of several iron-related genes, including *HvbHLH39, HvbHLH47, HvBGLU42, HvYSL1*, and *HvIRT1.* Surprisingly, the *HvMYB72* gene, an ortholog of *AtMYB72*, which is a key regulator in ISR and Fe-deficiency responses in Arabidopsis [23, 24], was not induced by beneficial bacteria in barley. The two plants use different strategies for iron uptake [61], which could be the reason for the different responses. Furthermore, the metal content of barley plants, including iron, copper, manganese, and zinc, was mildly enriched in the shoots upon inoculation with beneficial bacteria (Fig. 3F). These results indicate that Fe-deficiency responses were triggered by inoculation with beneficial bacteria rather than by nutrient deficiency. Very recently, the beneficial rhizobacterium *Bacillus velezensis* SQR9 has been demonstrated to cause root iron leakage through its type VII secretion system in the early stage after bacterial inoculation; in turn, the increased iron presence promoted bacterial colonization [62]. This offers a possible explanation for why iron homeostasis is regulated by bacteria during root colonization.

Another interesting point is that the antimicrobial compound coumarin (scopoletin) released from the roots of Arabidopsis can selectively inhibit certain soil-borne pathogens [63]. Meanwhile, coumarins are well-known phenolic compounds involved in iron uptake in plants [64]. In our study, *HvBGLU42*, an orthologous gene to *BGLU42* that plays a key role in the secretion of coumarins from roots to the rhizosphere in Arabidopsis [23], was highly induced by all three bacteria (Fig. 3C). Until now, no direct evidence was found to prove that barley can produce coumarins. A previous study reports that barley seedlings can take up umbelliferone, which is a natural product of the coumarin family, and modify it by methoxylation to yield scopoletin [65]. Furthermore, *Acidovorax radicis* N35 also influences flavonoid homeostasis in barley leaves upon *A. radicis N35* colonization of the root, decreasing the amount of lutonarin methylether [30]. Although phenolic compounds such as coumarin and flavonoids and their derivatives generally exist in parallel in plants, their multiple roles in plant development and defense must be considered [66]. On the other hand, canonical defense-related genes such as *HvPR1* and *HvPR17* were not regulated by bacterial inoculation in this study (Fig. 3D), which indicated that beneficial bacteria may trigger ISR through other pathways or other defense-related genes. Overall, the above information indicates that a common strategy linking the induction of ISR, response to Fe deficiency, and biosynthesis or metabolism of phenolic compounds such as coumarins seems possible.

Seed-associated microbiota play an important role in plant fitness. A recent study demonstrated that the seed microbiome was affected by soil fertility and beneficial bacteria when wheat seeds germinated in the corresponding field soil [67]. In this study, we present the first evidence that in barley, inoculation with beneficial bacteria of the parental plant (root drenching with *E. meliloti*) affected the composition of endophytes in harvested seeds (Fig. 4C). Moreover, our study indicates that, as reported previously, *Proteobacteria* are predominantly present in the seed microbiome (Fig. 4B) [68]. Interestingly, in seeds from field-grown barley plants, the number of ASVs related to the genus *Pantoea* increased after inoculation with *E. meliloti* (Fig. 4C), while this was not observed in seeds from greenhouse-grown barley plants (EF and PS, Fig. 4C). *Pantoea* was detected in seeds of field-grown barley plants on many occasions [31, 34]. Compared to greenhouse conditions, perhaps not surprisingly, field-grown plants produce seeds with more diverse microbiota. The high diversity of bacteria in air and water as well as contact with insects is a very probable explanation. Although inoculation with beneficial bacteria affected the seed endophytic microbiota, this phenomenon apparently did not lead to enhanced resistance in the next generation (Supplementary Fig. S1 and S2). In addition, the microbiota in both barley seeds and the rhizosphere are significantly influenced by the barley genotype [34]. Nevertheless, an enhancement of plant resistance through beneficial bacteria might be a good strategy in agriculture, even if this strategy requires further investigation.

## Conclusions

This study highlighted the interactions between different ISR-triggering bacteria and the crop plant barley. Our findings revealed that the ISR-triggering bacteria have different colonization patterns. Compared to the soil-borne *Ensifer meliloti*, *Pantoea* sp. and *Pseudomonas* sp., which were isolated from barley seeds, formed bacterial microcolonies in the maturation zone of the root or on root hairs, respectively.

Gene expression analysis identified genes related to iron homeostasis. These genes were differentially expressed after inoculation with *E. meliloti* and *Pantoea* sp., even if the host plants grew in an iron-sufficient environment and did not display Fe deficiency. This study revealed that an overlap between the Fe-deficient response and plant immunity response may exist in monocots, such as barley.

Translocation of bacteria from the root to the phyllosphere seems not to be a universal phenomenon. However, our study revealed that root colonization with beneficial bacteria influenced the composition of seed endophytes. Inoculation with *E. meliloti*, for example, increased the abundance of *Pantoea* sp. in seeds from field-grown plants. Further exploration of this phenomenon will help to fully use its benefits for crop plants.

## Materials and methods

### Plant material and growth conditions

Barley (*Hordeum vulgare* L.) cultivar Golden Promise (Simpsons Malt Limited, Berwick-upon-Tweed, United Kingdom) was used for all experiments. The surface sterilization of barley seeds was performed by using 2% sodium hypochlorite [34, 69]. Seeds were germinated on wet filter paper in the dark and at room temperature for 3 days and planted in standard potting substrate (Fruhstorfer Erde, Hawita Gruppe GmbH, Vechta, Germany). Plants were grown in a greenhouse at 18 °C with a 16/8-hour photoperiod (day/night).

### Regeneration of the endophyte-free barley plant

Barley (*Hordeum vulgare L.*) cultivar Golden Promise was grown in a climate chamber at 18 °C/14°C (light/dark) with 65% relative humidity, a 16 h photoperiod, and a photon flux density of 240 µmol m^− 2^ s^− 1^. Two weeks postanthesis, barley spikes were harvested. After removing the awns, kernels were put in a bottle and placed on ice. Approximately 100–200 kernels were surface sterilized in 70% ethanol for 5 min and subsequently incubated in sodium hypochlorite (3% active chlorine) for an additional 20 min. The kernels were washed once with sterilized water (pH 3) and then rinsed 3 times with sterile distilled water under sterile conditions. Immature embryos were extracted from the caryopses, and the embryonic axis was removed with a sharp scalpel using a binocular microscope. Immediately, the obtained scutella were placed onto a barley callus-inducing medium (BCID) composed of 4.3 g MS-stock, 1.2 mg CuSO_4_ × 5H_2_O, 30 g maltose, 1 mg thiamine HCl, 250 mg *myo*-inositol, 1 g casein hydrolysate, 690 mg L-proline and 2.5 mg dicamba in one L, adjusted to pH 5.8 and filter sterilized, then 6 g autoclaved phyto agar was added [38]. The calli were subcultured 3 times at an interval of 2 weeks under the same conditions and transferred to shoot- and root-inducing medium. Whole barley plantlets were generated as described by [37, 38].

### Bacterial cultivation

*Ensifer meliloti* (*Sinorhizobium meliloti*) strain Rm2011 was chosen because of its ability to produce a long-chain *N*-acyl-homoserine lactone, oxo-C14-HSL [35], which induces AHL-priming in many plants, including barley [19, 47, 70]. *Pantoea* sp. (strain P_s_AC_13b) and *Pseudomonas* sp. (strain P_s_CA_4b) were used as representative isolates from the barley seed core endophytes [31]. All three bacteria were marked with rifampicin resistance by cultivating the wild-type strains overnight on medium with rifampicin (50 µg/ml). The newly grown isolates were identified by box-PCR, with wild-type strains as controls. Bacterial strains with rifampicin resistance were used for the bacterial translocation assay. The plasmid pSM1890 encoding Green Fluorescent Protein (GFP) was inserted into the bacteria by performing triparental mating with a helper strain *Escherichia coli* CM544 R751 (incp-1 beta) and a donor strain *E. coli* CC118 lambda pir pSM1890. GFP-marked bacteria were used for localization studies. To test whether *Pantoea* sp. and *Pseudomonas* sp. produce AHL, the AHL biosensors *Chrornobacteriurn violaceum* Cv026 [71] and *C. violaceum* VIR07 [72] were used. Two known AHL producers, *Serratia plymuthica* HRO-C48 [73] and *Ensifer meliloti* (*Sinorhizobium meliloti*) strain Rm2011 [35], were used as positive controls. Control strains and tested bacteria were placed on an agar plate at two ends of *C. violaceum* cultures (Supplementary Fig. S3). The plates were incubated at 28 °C for 36 h. The biosensors produce violacein in response to AHL, displayed in a color change from white to purple. Bacteria were cultivated in tryptone yeast (TY) medium with the respective antibiotics rifampicin (50 µg/ml), streptomycin (250 µg/ml), and gentamycin (10 µg/ml).

### ISR assay

For the induced resistance assay, two-week-old barley was root-drenched with a bacterial suspension of 10^7^ colony forming units (CFU)/g soil, and 10 mM MgCl_2_ was used as a solvent control. For each bacterial treatment, inoculation was performed every third day for a total of three times.

Plant resistance to *Blumeria graminis* f. *sp. hordei* (*Bgh*) was assessed by counting fungal pustules in detached leaves of barley [41, 74]. Briefly, second and third leaves from inoculated or control barley plants were placed on water agar and challenged with fresh conidia (approximately 250 per cm^2^). Visible pustules were counted five days after challenge. Each treatment contained at least 130 leaves, and similar results were observed in three independent experiments.

### Determination of bacterial colonization patterns

For the determination of bacterial colonization patterns, plants were grown on sterile perlite supported with ¼ MS (Murashige and Skoog) medium in glass jars. Plants were inoculated with bacterial suspension (10^7^ CFU/ml) for three days.

Barley roots were gently washed with 10 mM MgCl_2_ from perlite and nonattached bacteria, stained with propidium iodide (PI) solution (1 µg/ml) for 5–10 min and subsequently mounted on a microscope slide in 4′,6-diamidine-2′-phenylindole dihydrochloride (DAPI) solution (10 µg/ml). Confocal laser scanning microscopy was performed using an SP8 confocal system (Leica Microsystems, Wetzlar, Germany) with three channels: excitation 405 nm, emission 430–480 nm (blue); excitation 488 nm, emission 500–550 nm (green); and excitation 561 nm, emission 600–680 nm (red), including autofluorescence.

### Bacterial translocation assays

To test whether bacteria translocate from the rhizosphere to the phylloplane, two-week-old barley plants were root-drenched with *Ensifer meliloti-*rif, *Pantoea* sp.*-*rif, and *Pseudomonas* sp.*-*rif (10^7^ CFU/g soil), 10 mM MgCl_2_ was used as a solvent control. The rhizosphere was sampled four hours, three days and seven days after drenching. The excess soil was removed from the roots by shaking. Then, entire roots were weighed and placed in 50 ml tubes with 9 ml of 10 mM MgCl_2_, followed by vortexing. The suspension was diluted with 10 mM MgCl_2_ in serial steps. Ten microliters were dropped on TY agar plates supplemented with rifampicin (50 µg/ml). Similarly, leaf samples were harvested at four hours, three days, and seven days after drenching. Approximately 1 cm of a leaf was cut off from plants and homogenized with 1 ml of 10 mM MgCl2. Ten microliters of the leaf sample’s original solution was dropped on TY agar plates supplemented with rifampicin (50 µg/ml). Each treatment was performed in four independent biological replicates.

### Transcriptome analysis

For the transcriptome analysis assay, the regenerated barley plants were treated with bacterial suspensions (10^7^ CFU/ml), and 10 mM MgCl_2_ was used as the solvent control. The samples were collected before (0 h) and 24 h after bacterial inoculation. Each treatment contained three independent biological replicates.

Total RNA was extracted with the RNeasy Plant Mini Kit (Qiagen, Germany). Library construction was performed by using the stranded mRNA enrichment method, and 20 M paired end (100 bp length) reads per sample were sequenced by Beijing Genomics Institute (BGI, China). The data analysis, including read mapping and feature counting, was performed within the R package Rsubread version 2.12.3 [75] with the default settings. The updated reference genome, MorexV3_pseudomolecules_assembly [76], was used for data analysis.

The identification of differentially expressed genes (DEGs) was performed by DESeq2 version 1.38.3 [77]. Samples were harvested at 0 h from the different independent jars to minimize the gene expression differences between individual plants. Meanwhile, treated samples at 24 h were normalized to samples at 0 h. The DEGs were identified by comparing expression between the bacterial treatment and solvent control using adjusted *p* < 0.05 and fold change > 1.5 as thresholds. To analyze gene functions, the protein sequence of identified DEGs was matched to the protein sequence of Arabidopsis (TAIR10) within the website tool BLAST search (https://plants.ensembl.org/tools.html). The analysis of the enriched gene ontology (GO) terms was performed with clusterProfiler version 4.6.2 [78]. Figures were created using R (version 4.2.1) and RStudio (“Ghost Orchid” Release).

### Quantitative reverse transcription PCR analysis

For quantitative RT-PCR, barley plants were grown on sterile perlite supported with ¼ MS (Murashige and Skoog) medium in glass jars. The plant roots were drenched with bacterial suspension (10^7^ CFU/g perlite). Samples were harvested from roots before and 24 h after bacterial inoculation. Each treatment was performed in four independent biological replicates.

Total RNA was extracted from plant samples using TriFast (peqGOLD, USA) and DNase I (Quanta Biosciences, USA) kits. cDNA synthesis was performed using the qScript cDNA Synthesis Kit (Quanta Biosciences, USA). The qRT-PCR reaction was run with the following program: initial denaturation at 95 °C for 60 s, denaturation at 95 °C for 15 s, and extension at 60 °C for 30 s with 40 cycles, and an additional melting curve from 60 to 95 °C. The expression of candidate genes was assessed with the primers listed in Supplementary Table S1.

### Metal content analysis of the bacteria-treated plants

For metal content analysis, sterile seedlings were grown in jars as described above for two weeks. The plant roots were drenched with bacterial suspension (10^7^ CFU/g perlite). Samples were harvested from the leaves and stems of seedlings 72 h after the bacterial treatment. Each treatment was performed in four independent biological replicates. Samples were dried in an oven at 70 °C and homogenized. Fifty milligrams of dried powder was mineralized with 2% nitric acid in borosilicate tubes using the ultraWAVE® system (Milestone, Italy) prior to ICP-MS quantification (iCAP-TQ, Thermo-Scientific). Oriental basma tobacco leaves (INCT-OBTL-5) and *Lemna minor* (BRC-670 Duck-Weed) were used as certified reference materials.

### Seed microbiota composition assay

To assess the seed microbial composition, three types of plants were tested: (i) endophyte-free barley plants inoculated with bacteria three days before being transferred into potting substrate and allowed to grow until harvest in the greenhouse; (ii) barley plants grown in potting substrate in the greenhouse until seed harvest; and (iii) barley plants grown at the field station in Julius Kühn Institute (Braunschweig, Germany). All three types of plants were inoculated with *E. meliloti* or control (10 mM MgCl_2_) three times. Seeds were harvested from parental plants after ripening, followed by extraction of total DNA.

To extract the total microbial DNA from barley seeds, 0.5 g of seeds was surface-sterilized and ground to powder. DNA was extracted with the FastDNA Spin Kit for Soil (MP Biomedicals, Eschwege, Germany). Amplicon sequencing libraries of the DNA samples were performed with a two-step PCR targeting the 16S rRNA gene’s V4 region, as described previously [41, 74, 79]. Sequencing was performed on an Illumina MiSeq platform with a Reagent Kit v2 (2 × 250 cycles) (Illumina, San Diego, CA, United States). The identification of amplicon sequence variants (ASVs) was performed by using the DADA2 version 1.10.0 plugin for QIIME2 (truncL = 0, truncR = 0; trimL = 8, trimR = 8, a minimum overlap of 12 bp) [79]. Each ASV sequence was annotated by using the *q2-feature-classifier* classifysklearn module trained with SILVA SSU rel. 132 database [80]. The final ASVs per sample were obtained by dividing each count of ASV per sample by the total amount of DNA per sample. Figure creation was performed by the ComplexHeatmap R package (version 2.8.0) [81] and BioRender (https://app.biorender.com).

### Statistical analysis

If not stated otherwise, statistical analysis was performed using R (version 4.2.1), and details are indicated in the figure legends. Quantitative RT-PCRs were performed with four independent biological replicates. ISR assays were performed in three biologically independent experiments. *p* values < 0.05 in Student’s *t*-test were considered indicative of a significant difference.

### Electronic supplementary material

Below is the link to the electronic supplementary material.

**Additional file 1**: **Supplementary Table S1**. List of primers used in this study.

**Additional file 2**: Supplementary Data Set S1. List of gene IDs and converted IDs

**Additional file 3**: **Supplementary Figure S1**. The enhanced resistance was lost in the next generation of greenhouse-grown barley. Barley seeds were harvested from endophyte-free (EF) and potting substrate barley (PS) plants inoculated with *E. meliloti*, *Pantoea* sp., *Pseudomonas* sp., or 10 mM MgCl_2_ as a control. The resistance against *Blumeria graminis* f. sp. *hordei* was assessed in the resulting plants. No differences were observed in the 2nd generation plants. Statistical analysis was performed with Student’s *t-*test, *n* = 50. **Supplementary Figure S2**. The enhanced resistance was lost in the next generation of field-grown barley. Barley seeds were harvested from field-grown (FG) barley plants inoculated with *E. meliloti* or 10 mM MgCl_2_ used as a control. The resistance against *Blumeria graminis* f. sp. *hordei* was assessed in the resulting plants. No differences were observed in the 2nd generation plants. Statistical analysis was performed with Student’s *t-*test, *n* = 50. **Supplementary Figure S3**. The AHL-biosensor strains *Chromobacterium violaceum* Cv026 and VIR07 respond to potential *N*-acyl homoserine lactone (AHL)-producing bacteria. *Serratia plymuthica* was used as a positive AHL-producing control for *C. violaceum* Cv026, and *E. meliloti* was used as a positive control for *C. violaceum* VIR07. The positive controls and the tested bacteria were placed at two ends of *C. violaceum* cultures. The biosensor color was evaluated after 36 h of cocultivation. Violacein of CV026 is inducible by AHL with *N*-acyl side chains from C4 to C8, whereas violacein production in VIR07 can be induced by long-chain AHL (C10–C16) but is inhibited by short-chain AHL (C4–C8). Violet coloration is indicative of AHL production.

## Data Availability

The raw sequences originating from the RNA-Seq approach and 16S rRNA gene amplicon sequencing can be accessed using the BioProject numbers PRJNA904663 and PRJNA904702 in the Sequence Read Archive (SRA), respectively.

## Abbreviations

AHL: *N*-acyl homoserine lactones
ASVs: Amplicon sequence variants
*Bgh*: Fungus *Blumeria graminis* f. sp. *hordei*
CFU: Colony forming units
DAPI: 4′,6-diamidine-2′-phenylindole dihydrochloride solution
DEGs: Differentially expressed genes
EF: Endophyte-free barley plants
EFE: Endophyte-free barley plants inoculated with *E. meliloti*
EFM: Endophyte-free barley plants inoculated with 10 mM MgCl_2_
FG: Field-grown plants
FGE: Field-grown plants inoculated with *E. meliloti*
FGM: Field-grown plants inoculated with 10 mM MgCl_2_
GFP: Green Fluorescent Protein
GO: Gene ontology terms
MS: Murashige and Skoog medium
PGPB: Plant growth-promoting bacteria
PI: Propidium iodide solution
PS: Potting substrate barley plants
PSE: Potting substrate barley plants inoculated with *E. meliloti*
PSM: Potting substrate barley plants inoculated with 10 mM MgCl_2_
ISR: Induced systemic resistance
TY: Tryptone yeast medium
