# Supplementary material for: Hordeum vulgare differentiates its response to beneficial bacteria
Source: BMC Plant Biol. 2023 Oct 4;23:460. doi: 10.1186/s12870-023-04484-5 (PMC10548682; doi:10.1186/s12870-023-04484-5)
Supplement: Supplementary file 1 — Additional file 1: Supplementary Table S1. List of primers used in this study. [file 12870_2023_4484_MOESM1_ESM.docx]

## Supplementary Table S1. List of primers used in this study

| **gene name** | **primer name** | **Sequence (5'->3')** |
| --- | --- | --- |
| *HvIRT1* | *RT-hvIRT1-1F* | CCATGCTCTTGCCAGTTTGG |
|  | *RT-hvIRT1-1R* | GGGAAAGCTGCAACTCGGTA |
| *HvYSL1* | *RT-YSL1-1F* | CCAGACCGCAGCCTACTAAG |
|  | *RT-YSL1-1R* | TACCTATTGGCTCCTGCCCT |
| *HvMYB72* | *HvMYB72_1F* | AAGAACGTCTGGCACACACA |
|  | *HvMYB72_1R* | CCAACGACGAGGCTACTGAG |
| *HvBGLU42* | *HvBGLU42_2F* | TACCCATCTCACATGCCCTTC |
|  | *HvBGLU42_2R* | AGCAGCAGCTAAGTAGCACAC |
| *HvbHLH39(ORG3)* | *HvbHLH39_F* | ACAGTGCTGCGTGTTCCATA |
|  | *HvbHLH39_R* | CCCGGCCTGTGCATCAATA |
| *HvbHLH47(PYE)* | *HvbHLH47_1F* | TGAGCTGCAGGATGAGAACG |
|  | *HvbHLH47_1R* | TGTGACGGGAAAACTGTGCT |
| *HvPR1* | *HvPR1_F* | GGACTACGACTACGGCTCCA |
|  | *HvPR1_R* | GGCTCGTAGTTGCAGGTGAT |
| *HvPR17* | *HvPR17_F* | CGAGGTTCCTCGACTACTGC |
|  | *HvPR17_R* | ATCACATTCAGCCTCCGAAC |
| *HvHSP70* | *HvHsp70_F* | CCAAGAAGTCGCAGGTTTTC |
|  | *HvHsp70_R* | GGAATGCCAGAAAGGTCAAA |
| *HvUBQ60* | *HvUBQrev* | CAGTAGTGGCGGTCGAAGTG |
|  | *HvUBQfrw* | ACCCTCGCCGACTACAACAT |
