# Supplementary material for: Hordeum vulgare differentiates its response to beneficial bacteria
Source: BMC Plant Biol. 2023 Oct 4;23:460. doi: 10.1186/s12870-023-04484-5 (PMC10548682; doi:10.1186/s12870-023-04484-5)
Supplement: Supplementary file 3 — Additional file 3: Supplementary Figure S1. The enhanced resistance was lost in the next generation of greenhouse-grown barley. Barley seeds were harvested from endophyte-free (EF) and potting substrate barley (PS) plants inoculated with E. meliloti, Pantoea sp., Pseudomonas sp., or 10 mM MgCl2 as a control. The resistance against Blumeria graminis f. sp. hordei was assessed in the resulting plants. No differences were observed in the 2nd generation plants. Statistical analysis was performed with Student’s t-test, n = 50. Supplementary Figure S2. The enhanced resistance was lost in the next generation of field-grown barley. Barley seeds were harvested from field-grown (FG) barley plants inoculated with E. meliloti or 10 mM MgCl2 used as a control. The resistance against Blumeria graminis f. sp. hordei was assessed in the resulting plants. No differences were observed in the 2nd generation plants. Statistical analysis was performed with Student’s t-test, n = 50. Supplementary Figure S3. The AHL-biosensor strains Chromobacterium violaceum Cv026 and VIR07 respond to potential N-acyl homoserine lactone (AHL)-producing bacteria. Serratia plymuthica was used as a positive AHL-producing control for C. violaceum Cv026, and E. meliloti was used as a positive control for C. violaceum VIR07. The positive controls and the tested bacteria were placed at two ends of C. violaceum cultures. The biosensor color was evaluated after 36 h of cocultivation. Violacein of CV026 is inducible by AHL with N-acyl side chains from C4 to C8, whereas violacein production in VIR07 can be induced by long-chain AHL (C10–C16) but is inhibited by short-chain AHL (C4–C8). Violet coloration is indicative of AHL production. [file 12870_2023_4484_MOESM3_ESM.pdf]

Supplementary Fig. S1

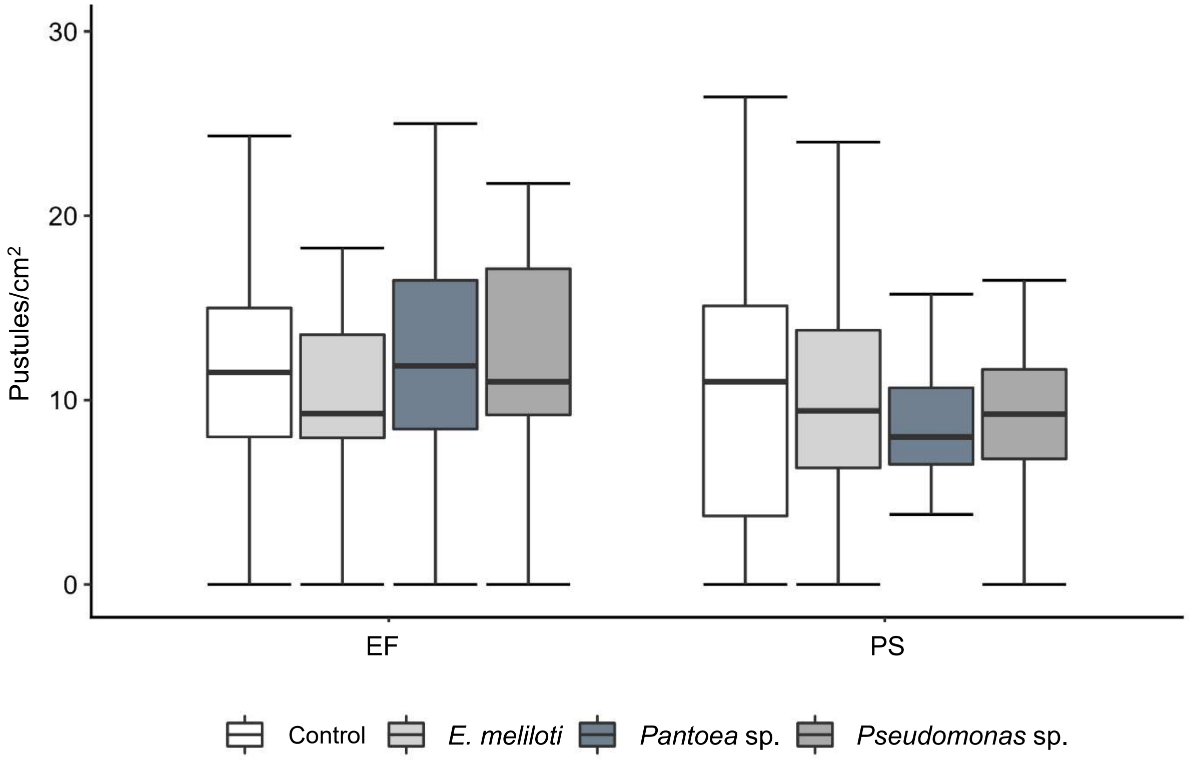

Supplementary Figure S1. The enhanced resistance was lost in the next generation of greenhouse-grown barley

Barley seeds were harvested from endophyte-free (EF) and potting substrate barley (PS) plants, inoculated with *E. meliloti*, *Pantoea* sp., *Pseudomonas* sp., or 10 mM $MgCl_2$ as a control. The resistance against *Blumeria graminis* f. sp. *hordei* was assessed in the resulting plants. No differences were observed in the 2nd generation plants. Statistical analysis was performed with Student's *t*-test, *n* = 50.

Supplementary Fig. S2

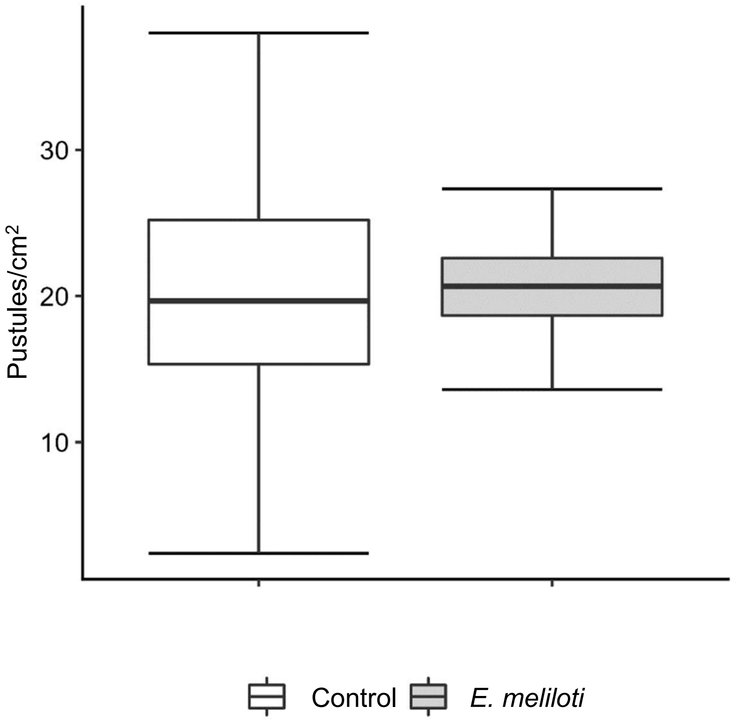

Supplementary Figure S2. The enhanced resistance was lost in the next generation of field-grown barley

Barley seeds were harvested from field-grown (FG) barley plants inoculated with *E. meliloti* or 10 mM $MgCl_2$ used as a control. The resistance against *Blumeria graminis* f. sp. *hordei* was assessed in the resulting plants. No differences were observed in the 2nd generation plants. Statistical analysis was performed with Student's *t*-test, *n* = 50.

Supplementary Fig. S3

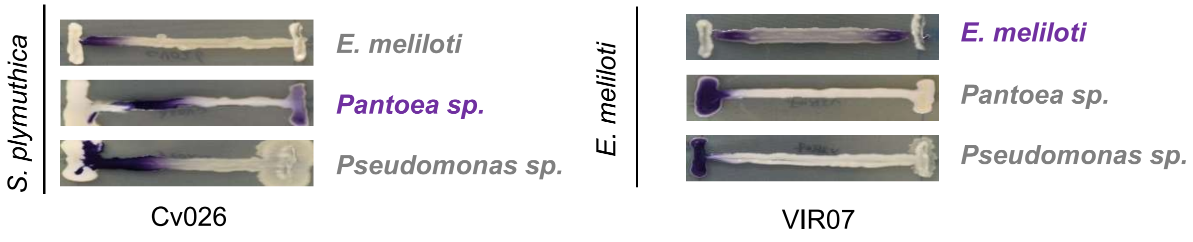

Supplementary Figure S3. The AHL-biosensor strains *Chromobacterium violaceum* Cv026 and VIR07 respond to potential *N*-acyl homoserine lactone (AHL)-producing bacteria

Seratia plymuthica was used as a positive AHL-producing control for *C. violaceum* Cv026 and *E. meliloti* was used as a positive control for *C. violaceum* VIR07. The positive controls and the tested bacteria were placed at two ends of *C. violaceum* culture. The biosensor color was evaluated after 36 h of cocultivation. Violacein of CV026 is inducible by AHL with *N*-acyl side chains from C4 to C8, whereas violacein production in VIR07 can be induced by long-chain AHL (C10–C16), but is inhibited by short-chain AHL (C4–C8). Violet coloration is indicative of AHL production.
